# Activation of STAT3 Regulates Reactive Astrogliosis and Neuronal Death Induced by AβO Neurotoxicity

**DOI:** 10.3390/ijms21207458

**Published:** 2020-10-10

**Authors:** Danira Toral-Rios, Genaro Patiño-López, Gisela Gómez-Lira, Rafael Gutiérrez, Fernando Becerril-Pérez, Aldebarán Rosales-Córdova, Juan Carlos León-Contreras, Rogelio Hernández-Pando, Ismael León-Rivera, Isabel Soto-Cruz, Benjamín Florán-Garduño, Victoria Campos-Peña

**Affiliations:** 1 Departamento de Fisiología, Biofísica y Neurociencias, Centro de Investigación y de Estudios Avanzados del Instituto Politécnico Nacional, Ciudad de Mexico 07360, Mexico; dtoral@fisio.cinvestav.mx (D.T.-R.); bfloran@fisio.cinvestav.mx (B.F.-G.); 2 Laboratorio de Investigación en Inmunología y Proteómica, Hospital Infantil de México Federico Gómez, Ciudad de Mexico 06720, Mexico; gena23pat@yahoo.com; 3 Departamento de Farmacobiología, Centro de Investigación y de Estudios Avanzados del Instituto Politécnico Nacional, Ciudad de Mexico 14330, Mexico; glira@cinvestav.mx (G.G.-L.); rafagut@cinvestav.mx (R.G.); 4 Research Institute of Molecular Pathology (IMP), Vienna Biocenter (VBC), Campus-Vienna-BioCenter 1, 1030 Vienna, Austria; fernando.becerril@imp.ac.at; 5 Departamento de Administración, Facultad de Economía y Negocios, Universidad Anáhuac de México, Huixquilucan 52786, Mexico; carlos.rosalesc@anahuac.mx; 6 Departamento de Patología, Sección Patología Experimental, Instituto Nacional de Ciencias Médicas y Nutrición, Salvador Zubirán, Ciudad de Mexico 14080, Mexico; jcleonc@hotmail.com (J.C.L.-C.); rhdezpando@hotmail.com (R.H.-P.); 7 Centro de Investigaciones Químicas, IICBA, Universidad Autónoma del Estado de Morelos, Cuernavaca Morelos 62210, Mexico; ismaelr@uaem.mx; 8 Laboratorio de Oncología Molecular, Facultad de Estudios Superiores Zaragoza, Universidad Nacional Autónoma de México, Ciudad de Mexico 09230, Mexico; sotocruz@unam.mx; 9 Laboratorio Experimental de Enfermedades Neurodegenerativas, Instituto Nacional de Neurología y Neurocirugía Manuel Velasco Suárez, Ciudad de Mexico 14269, Mexico

**Keywords:** β-amyloid, oligomers, neurotoxicity, neuroinflammation, Alzheimer’s disease, STAT3

## Abstract

Amyloid-beta oligomers (AβO) have been proposed as the most potent neurotoxic and inflammation inducers in Alzheimer’s disease (AD). AβO contribute to AD pathogenesis by impairing the production of several cytokines and inflammation-related signaling pathways, such as the Janus kinases/signal transducer of transcription factor-3 (JAK/STAT3) pathway. STAT3 modulates glial activation, indirectly regulates Aβ deposition, and induces cognitive decline in AD transgenic models. However, in vivo studies using an AβO microinjection rat model have not yet explored STAT3 role. The main purpose of this study was to elucidate if a single microinjection of AβO could promote an increased expression of STAT3 in glial cells favoring neuroinflammation and neurodegeneration. We designed a model of intrahippocampal microinjection and assessed glial activation, cytokines production, STAT3 expression, and neurodegeneration in time. Our results showed robust expression of STAT3 in glial cells (mainly in astrocytes) and neurons, correlating with neuronal death in response to AβO administration. A STAT3 inhibition assay conducted in rat primary hippocampal cultures, suggested that the induction of the transcription factor by AβO in astrocytes leads them to an activation state that may favor neuronal death. Notwithstanding, pharmacological inhibition of the JAK2/STAT3 pathway should be focused on astrocytes because it is also essential in neurons survival. Overall, these findings strongly suggest the participation of STAT3 in the development of neurodegeneration.

## 1. Introduction

Alzheimer’s disease (AD) is a neurodegenerative condition characterized by the presence of intracellular neurofibrillary tangles and extracellular neuritic plaques (NP), induced by beta-amyloid (Aβ) peptide accumulation. For years, the Aβ aggregation hypothesis has been proposed as the central mechanism underlying the development of the disease, yet it is still controversial [1,2,3]. Aβ peptides of 37–42 amino acids are released after the proteolytic cleavage of the trans-membrane amyloid β precursor protein (AβPP) by the β-secretase enzyme and the γ-secretase complex [4,5,6]. Despite Aβ40 and Aβ42 being the main NP components, Aβ42 seems responsible for the accelerated peptide nucleation and deposition [7,8]. In general, NPs consist of insoluble Aβ fibrils [9,10]. However, several studies have shown that a wide variety of soluble oligomers are highly toxic, and it has been suggested the existence of an inverse correlation between oligomer size and toxicity [11], Aβ neurotoxicity depends on primary structure and aggregation state. Several reports have indicated that high molecular weight oligomers alter the integrity of the membrane by favoring the generation of ROS and lipid peroxidation. This leads to a decrease in membrane fluidity, intracellular calcium dysregulation, depolarization, and impaired LTP [12]. In contrast, dimers of Aβ are related to Tau phosphorylation, and astrocyte and microglial activation [13]. Finally, AβO cause synaptic plasticity dysfunction, cognitive impairment, and cell death through the extracellular interaction with the N-methyl-d-aspartate receptor (NMDAR) [14,15,16].

Neuroinflammation is another indirect mechanism of Aβ fibrils and oligomers that leads to neurotoxicity and precedes cognitive decline. Aβ is recognized through different receptors located in glial cells, such as the T-cell ligand receptors, NOD-like receptors, formyl peptide receptors, and scavenger receptors [17]. This recognition triggers the clearance of Aβ from the central nervous system (CNS) by the glial cells [18], and activates different signaling pathways to favor the production and release of several proinflammatory mediators like the interleukin-1 beta (IL-1β), interleukin-6 (IL-6), tumor necrosis factor-alpha (TNF-α), and interferon-gamma (INF- γ). These mediators condition the glial immune response to an A1/M1 phenotype, favoring phagocytosis [19], reactive oxygen species generation, apoptosis, inflammasome stimulation, Aβ overproduction in astrocytes, tau phosphorylation, and neuronal damage [20,21,22,23,24]. 

Several models have assessed the AβO toxic effects. Studies using astrocytes and microglial cultures have revealed that AβO are potent proinflammatory inducers in glial cells in comparison to fibrils, but the latter could be responsible for chronic inflammation establishment [25,26]. Nevertheless, astrocytes and microglia can release other molecules that switch on neurodegenerative mechanisms related to chronic inflammation.

Signal transducers and transcription activators (STAT) are a family of cytoplasmic factors activated by phosphorylation through the Janus kinases (JAK) pathway, primed by several cytokines. Cytokines such as IL-6 induce the JAK2/STAT3 pathway and regulate several transcription factors linked to inflammation [27]. Furthermore, the JAK/STAT pathway immunomodulatory roles have been associated to neurodegenerative diseases, including AD. According to the above, Aβ induces STAT3 activation in neurons favoring cell damage and death [28]. Other studies have proposed that STAT3 activation in astrocytes modifies Aβ clearance or deposition, inflammation, and synaptic disruption [29,30]. Although microglial STAT3 activation is still poorly documented, it has been suggested that STAT3 phosphorylation could be an initial activation event [31].

We investigated the effects of AβO on neurodegeneration linked to neuroinflammation along 18 h, 72 h, and 7, 15 and 30 days, after its intrahippocampal microinjection. We aimed to identify STAT3 expression, and our findings suggest an increase of STAT3 expression in glial cell, induced by AβO. For this, two JAK2/STAT3 inhibitors were tested in primary cell culture, demonstrating the relevance of the pathway in astrocytes activation, a process linked to neurodegeneration development. The JAK2/STAT3 pathway modulation could be a promising strategy to control neuronal death, only directed towards astrocytes because if it carried out in neurons could contribute neurodegeneration. Future pharmacological research could focus on the modulation of the signaling pathways that regulate the glial phenotype, this would allow elucidating the neuroinflammation and neurodegeneration process present in AD. 

## 2. Results

### 2.1. Aβ-Mediated Neuronal Degeneration

**Figure 1:**
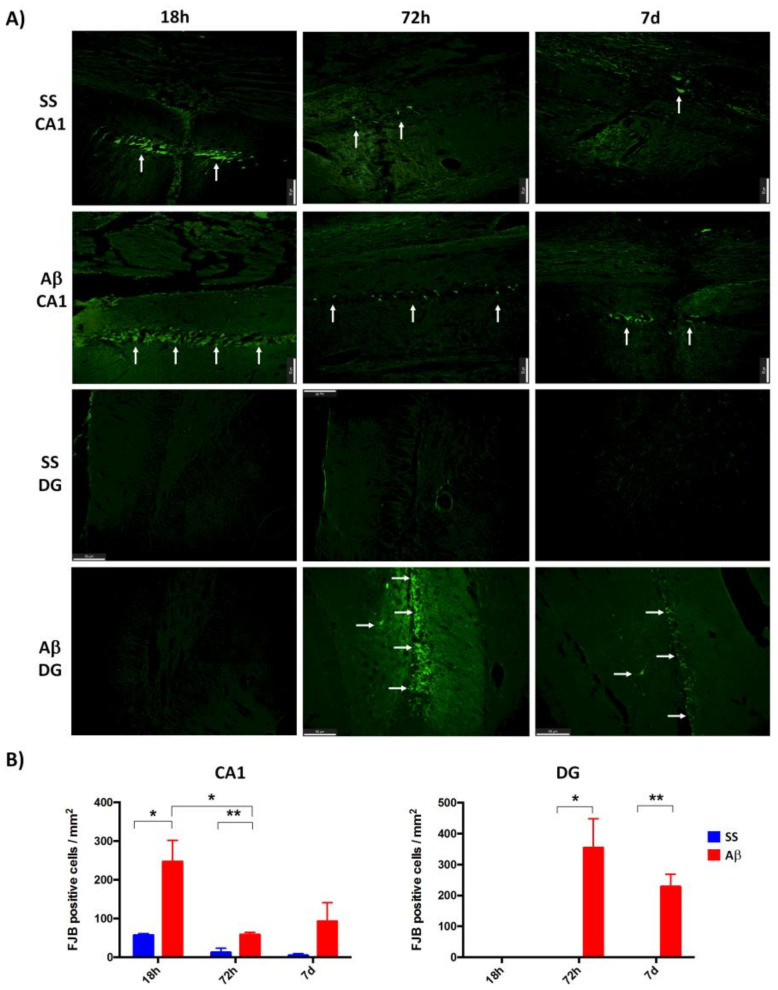
Quantification of degenerated neurons. FJB staining was used to identify neurons undergoing neurodegeneration in CA1 and DG. (**A**) Representative images (20×) of SS and Aβ rats sacrificed after 18 h, 72 h and 7 days are shown (Scale bar = 50 µm, white arrows mark FJB positive cells). Aβ oligomers promote increased cell death in both regions more than SS. (**B**): Quantitative analysis (*n* = 3) in CA1 show a higher FJB stain in oligomers rats at 18 h and decreased at 72 h. Meanwhile, in DG, FJB positive cells were only detected in Aβ rats sacrificed after 72 h. Data of FJB positive cells/mm^2^ are presented as mean with the standard error of mean (SEM) bars. Significance level * *p* < 0.05 and ** *p* < 0.01, are marked.

Neuronal degeneration was evaluated with the Fluoro-Jade B (FJB) staining in rats sacrificed at 18 h, 72 h, and 7, 15, and 30 days after AβO microinjection. As shown in Figure 1A, AβO promoted a significant increase of FJB positive cells (*p* < 0.05), compared with saline solution treatment at 18 h in the CA1 region. After 72 h, the number of FJB positive cells decreased in AβO and SS groups, remaining discretely higher (*p* < 0.05) in rats injected with AβO. Although the CA1 region showed an apparent increase of FJB cells in the AβO group at day 7, the difference was not significant. In contrast, FJB positive cells were evident in the dentate gyrus (DG) region of AβO microinjected rats sacrificed at 72 h and 7 days post-injection. Even though there is an apparent reduction of FJB cells in this time window, the statistic analysis did not show differences between the two sacrifice time points.

**Figure 2:**
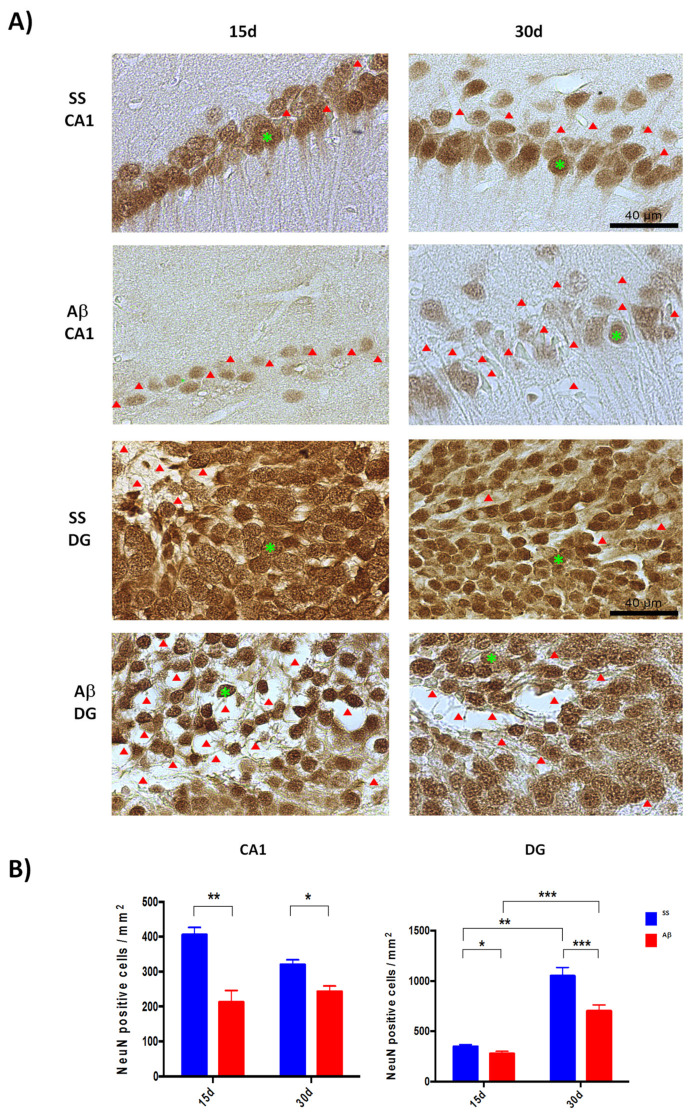
Quantification of mature neurons. Brains sections were immuno-stained for NeuN. CA1 and DG regions of rats sacrificed 15 and 30 days after SS or Aβ microinjection were evaluated. (**A**): Representative 40× images show that Aβ oligomers favor neuron loss in both regions (a green asterisk point one NeuN positive cell in each image, red arrowheads represent spaces of apparent neuronal loss). (**B**): Quantitative analysis (*n* = 3) suggests a recovery of NeuN positives cells in DG between 15 and 30 days. Data of NeuN positive cells/mm^2^ are presented as mean with SEM bars. Significance level * *p* < 0.05, ** *p* < 0.01, and *** *p* < 0.001 are marked.

The FJB stain decreased at 15 and 30 days, after microinjection of AβO (data not shown). For this reason, we quantified the neuronal nuclei protein (NeuN) positive cells in the hippocampus (Figure 2A), at these two time points, a reduction of NeuN positive cells in CA1 after AβO injection was evident at both times as compared with the control group (SS) (*p* < 0.05) (Figure 2B). 

Furthermore, immunohistochemistry (IHC) evidenced neuronal death, some dystrophic neurites and pyramidal cell disarray promoted mostly by AβO compared with the SS injection. Also, we found a moderate de crease of NeuN positive cells between 15 and 30 days in control groups (*p* < 0.05). AβO-injected rats showed a higher neuronal loss over time. The spaces in the preparations left by dead cells were detected mainly in DG of rats treated with oligomers and sacrificed at day 15, correlating with a significant decrease of NeuN positive cells in AβO group (*p* < 0.05) (Figure 2B). Such neuronal loss was less evident after 30 days of oligomers microinjection, in agreement with an increase of NeuN positive cells (*p* < 0.05). This observation may be related to a recovery phenomenon in the DG region [32], which is a neurogenesis niche. 

### 2.2. Acute and Chronic Cellular Response Mediated by Aβ Oligomers

A theory for AβO toxicity is by means of glial activation, which induces an inflammatory microenvironment and thus neurodegeneration. We therefore sought to identify the presence of mature neurons with an anti-NeuN antibody, microglial cells with an anti-Ionized calcium-binding adapter molecule 1 (Iba1) antibody, and an anti-glial fibrillary acidic protein (GFAP) antibody to detect astrocytes.

**Figure 3:**
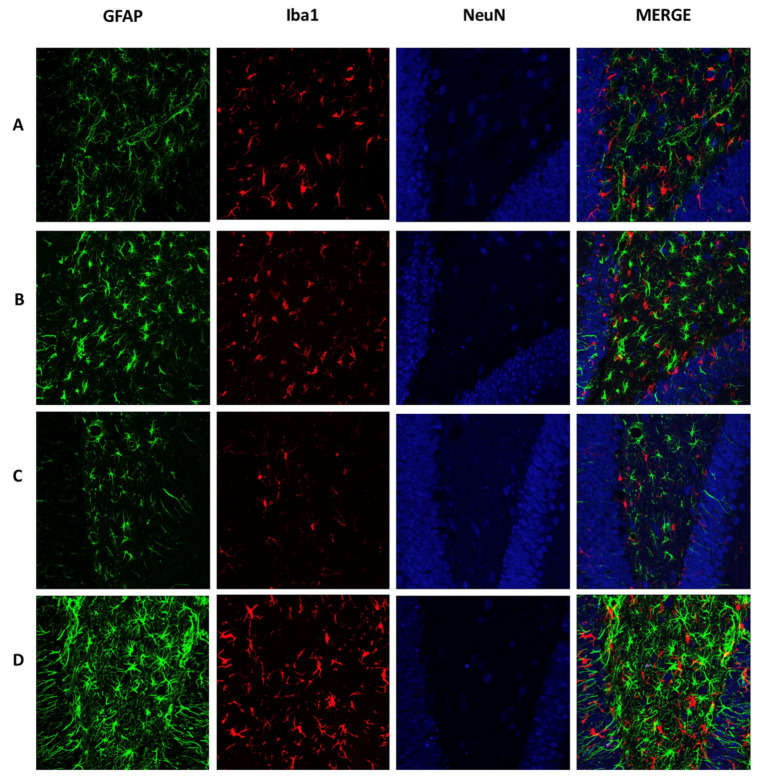
Acute cellular response mediated by AβO. Brain sections were immunolabeled to detect mature neurons (Anti-Neun, Cy5), astrocytes (Anti-GFAP, Alexa 488), and microglia cells (Anti-Iba, Alexa 555). Representative images (40×) of DG show the cellular response in the groups: (**A**) SS 18 h, (**B**) Aβ 18 h, (**C**) SS 72 h, and (**D**) Aβ 72 h. AβO treatments (**B** and **D**) increase astrocytes and microglial cells, as well as neuronal density in DG.

In Figure 3, images from the acute cellular response in the DG show that, at 18 h, AβO (Figure 3B) promotes an increase of GFAP and Iba1 immunoreactivity, while NeuN cells showed a slight reduction compared with the saline solution group (Figure 3A). The mechanic injury by itself increased astrocytes and microglial cells in the control group. However, in the AβO group, we found a high number of swollen ramified Iba1 cells and few with spherical morphology. We also found an increased number of GFAP cells with hypertrophy of body and stem processes. These morphological changes are signs of glial activation. After 72 h, GFAP and Iba1 staining decreased in the SS group (Figure 3C) in comparison with the previous time point. Interestingly, AβO maintains the number of GFAP and Iba1 cells, preserving the activated morphology (Figure 3D). These changes relate to a reduction of DG density and NeuN staining.

**Figure 4:**
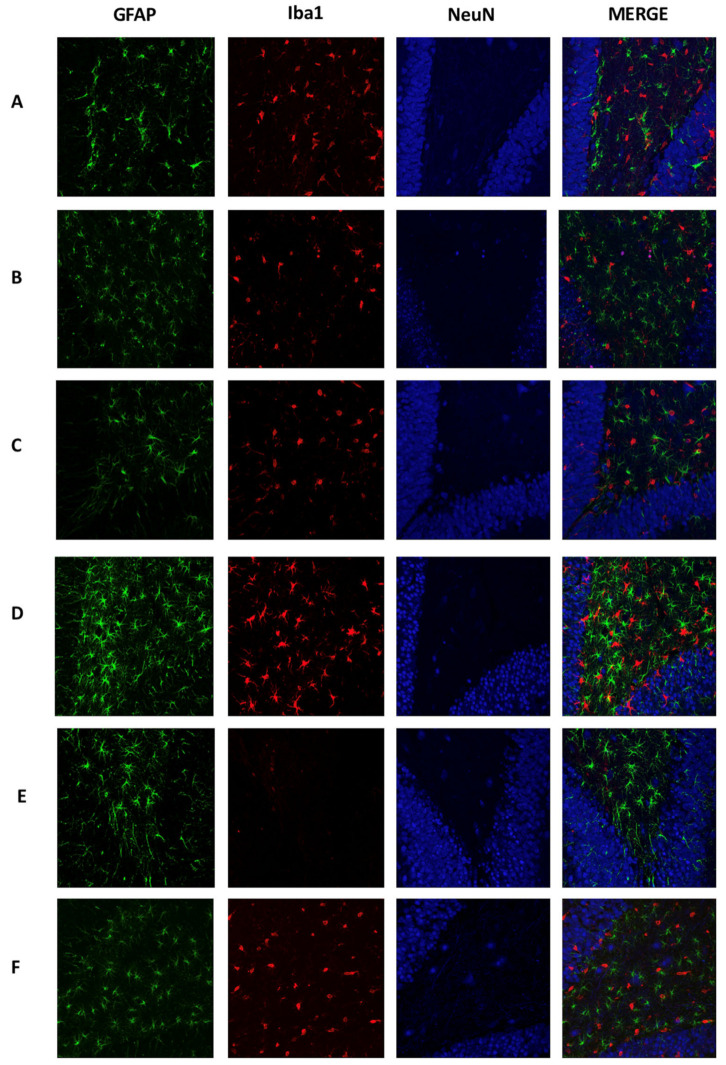
Chronic cellular response mediated by AβO. Brain sections were immunolabeled to detect mature neurons (Anti-Neun, Cy5), astrocytes (Anti-GFAP, Alexa 488), and microglia cells (Anti-Iba, Alexa 555). Representative 40× images of DG show cellular response in the groups: (**A**) SS 7 d, (B) Aβ 7 d, (**C**)SS 15 d, (**D**) Aβ 15 d, (**E**) SS 30 d, and (**F**) Aβ 30 d. Hypertrophic astrocytes were higher in AβO-injected rats (**B**, **C**, and **D**) with concomitant activation of microglial cells and a reduction of the neuronal density in DG.

The immunofluorescence at day 15 showed a reduction of astrocyte hypertrophy in the control group as compared to day 7 (Figure 4C). Similarly, the number of NeuN positive cells was reduced significantly in AβO microinjected rats (Figure 4D). Also, the number of hypertrophic astrocytes and swollen ramified microglial cells was doubled on day 15. Some of these Iba1 cells are close to neuronal loss spaces (Figure 4D). The control group (Figure 4E) showed a reduction in the number of Iba1 positive cells while AβO promoted a higher number of Iba1 positive cells with spherical or amoeboid morphology at day 30 (Figure 4F). Even though GFAP staining in the AβO group is similar to that in control, we found differences in the astrocyte´s morphology of oligomer-injected rats (hypertrophy). Additionally, NeuN staining decreased in comparison to the control group, but neuronal loss spaces were fewer compared to the observed at day 15. This result can also relate to a recovery phenomenon proposed in the NeuN quantification between 15 and 30 days.

### 2.3. Ultrastructural Analysis

**Figure 5:**
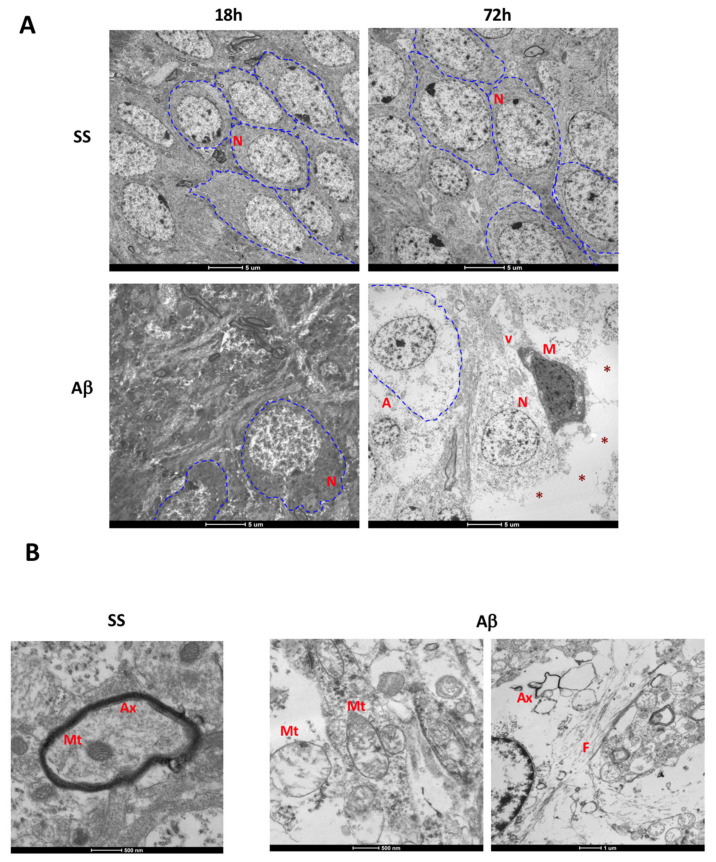
Acute ultrastructural analysis. (**A**) Transmission electron micrographs (Scale bar: 5 µm) of 18 h and 72 h are shown. Blue dashed lines identify at least one neuron (N) in each image. After 18 h, AβO decreases neuron density. At 72 h, neuronal loss spaces are evident (red asterisks), in addition to swollen astrocytes (A), and the presence of vacuolated microglial cells (M). (**B**) Magnification at 72 h show the presence of swollen mitochondria (Mt), axon (Ax) degeneration, vacuoles in the neuropil, and fibrils (F) in the AβO group.

Transmission electron microscopy allowed us to analyze the time course of ultrastructural changes induced by AβO. AβO favored neuronal loss in rats at 18 h when compared to the control group, where the cellular structure was conserved as shown with FJB staining (Figure 5A).

Despite the mechanic injury in the control group, cellular ultrastructure and density remained conserved after 72 h. Edema and vacuoles in the neuropil were evident in oligomer-treated rats, suggesting neuronal loss. Also, neurons suffered severe cytoplasm disruption followed by loss of organelles, such as endoplasmic reticulum and mitochondria. Moreover, we detected swollen astrocytes and vacuolated microglial cells surrounding neurons on the degeneration process, correlating with the activated morphology observed by immunofluorescence. Other observations at this time after AβO injection were the presence of swollen mitochondria, axon degeneration, and amyloid-like fibrils, whereas these were absent in the control group (Figure 5B). 

**Figure 6:**
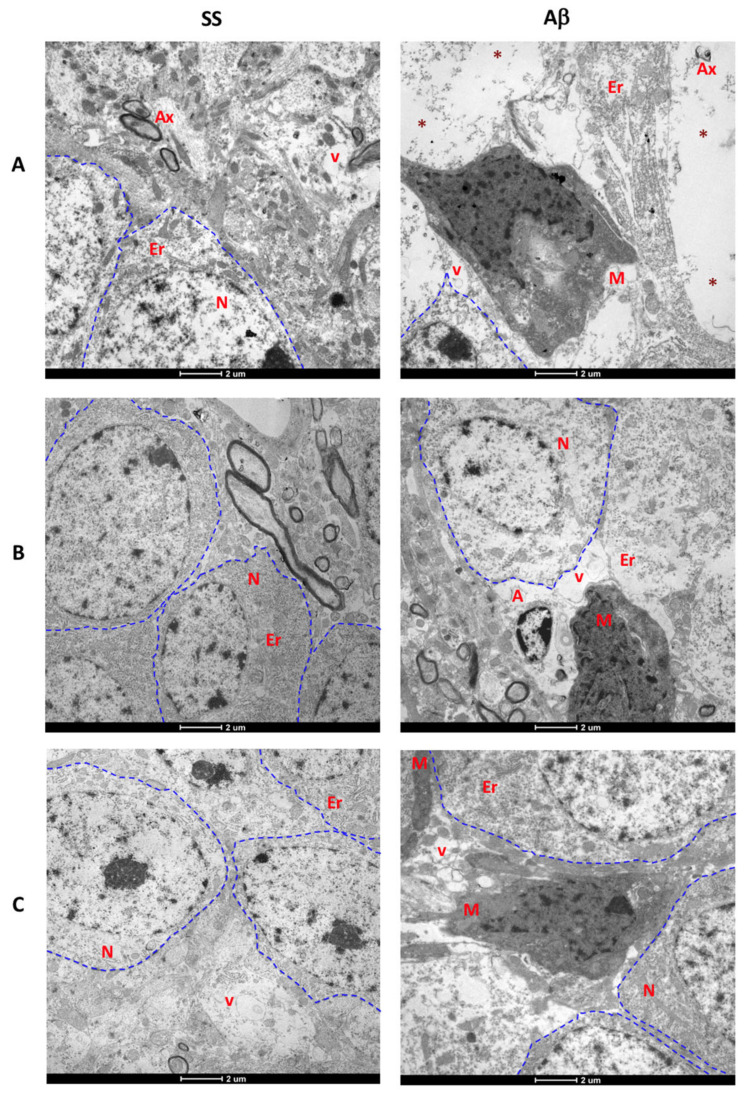
Ultrastructural analysis in chronic days. Transmission electron micrographs (Scale bar: 2 µm) of (**A**) 7 d, (**B**) 15 d, and (**C**) 30 d are shown. Blue dashed lines identify at least one neuron (N) in each image. In general, Aβ oligomers induce microglial (M) activation, disruption of cytoplasm and endoplasmic reticulum (ER) in rats sacrificed at 7, 15, and 30 days after injection. At day 7, oligomers promote an increased number of vacuoles (v) in the neuropil and neuronal loss spaces (red asterisks). These spaces are less evident in AβO rats sacrificed at day 15, but swollen astrocytes (A) and vacuolated microglial cells were found. At day 30 some of these cells were still present, but neuron loss, cytoplasm disruption, and alterations in endoplasmic reticulum were less evident.

**Figure 7:**
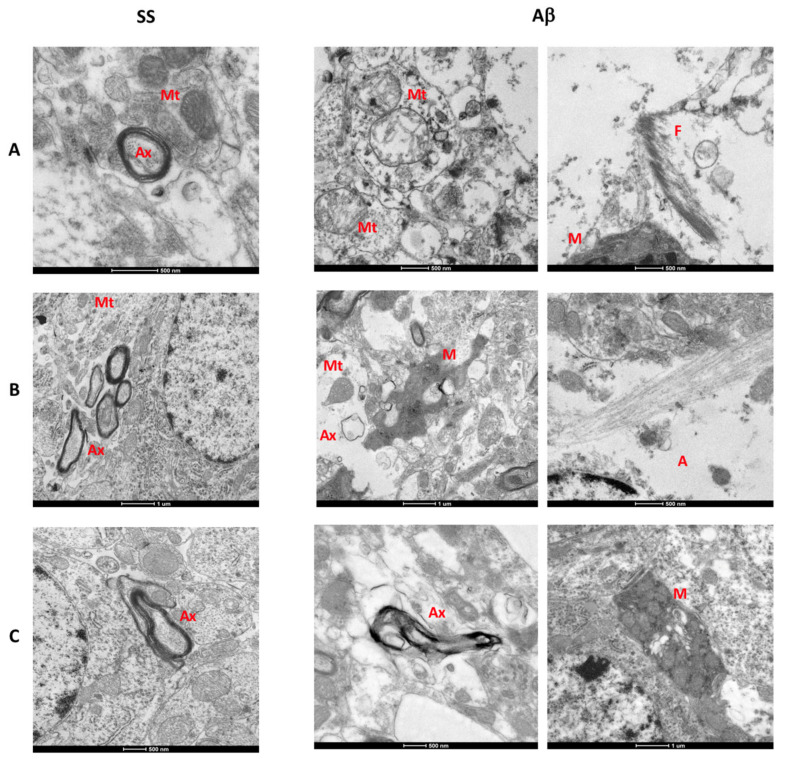
Additional ultrastructural abnormalities. Electron micrographs (Different scale bars) of (**A**) 7, (**B**) 15, and (**C**) 30 days are shown. Fibrils (F), axon (Ax) degeneration, mitochondrial (Mt) abnormalities and vacuolated microglial (M) cells are present in rats sacrificed after 7 and 15 days of AβO injection. At day 15, we observed microglial processes with vacuoles and swollen astrocytes (A). At day 30 post-injection, cytoplasm disruption, and neuronal loss decreased, but few activated microglia and axon degeneration persisted.

At day 7, the AβO group showed the presence of vacuoles in the neuropil, endoplasmic reticulum disruption, axonal degeneration, abundant neuronal loss spaces, vacuolated microglia, and cytoplasm disruption (Figure 6A). In addition to these changes, swollen mitochondria and activated microglia close to amyloid-like fibrils were observed (Figure 7A). The control group only exhibited vacuoles in the neuropil.

After 15 days, we found an apparent structural recovery in the SS and AβO rats (Figure 6B). The control group showed greater preserved neurons than control animals sacrificed on day 7. In contrast, the 15 day-AβO rats exhibited vacuoles and disruption of ER and cytoplasm, in addition to swollen astrocytes and vacuolated microglia. Amplified images (Figure 7B) showed axon degeneration, vacuolated microglial processes, and decreased mitochondrial dysfunction.

Interestingly at day 30, microstructural recovery was observed in the cytoplasm and mitochondria of neurons in AβO-injected rats. However, activated microglia and axonal degeneration persisted, none of these changes were found in the control group (Figure 6C and Figure 7C).

### 2.4. Molecular Inflammation Mediated by Aβ Oligomers

**Figure 8:**
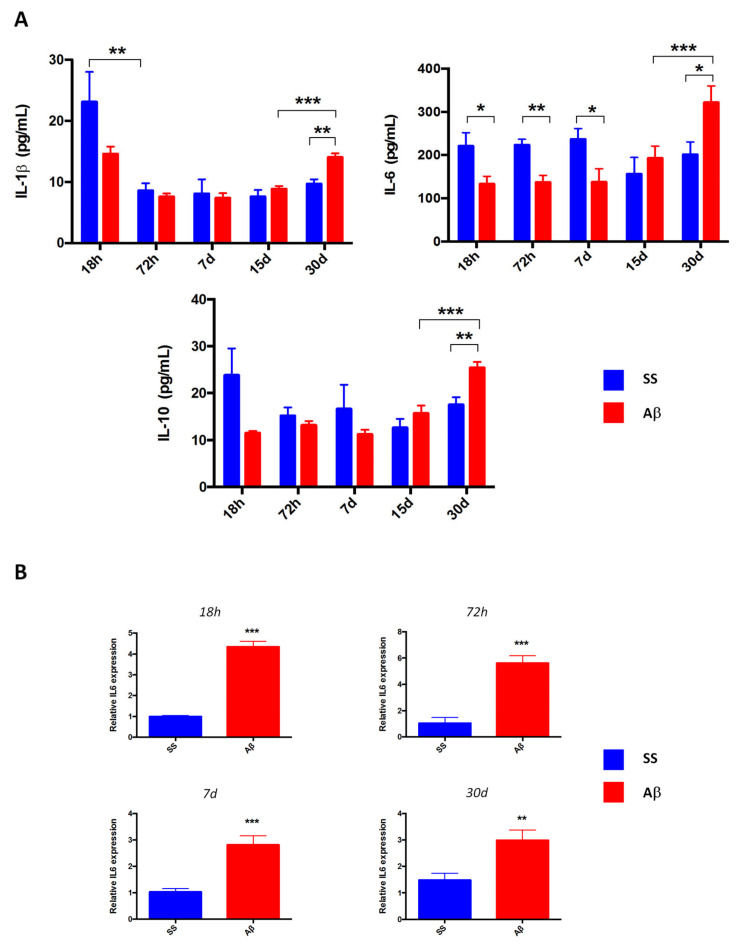
Cytokine quantification and IL-6 expression. (**A**) Quantitative analysis in hippocampus homogenates (*n* = 4) reveals an increased release of IL-1β, IL-6, and IL-10 after 30 days of AβO microinjection. Interestingly, IL-6 decreased in Aβ groups sacrificed after 18 h, 72 h and 7 days. (**B**) qPCR analysis (*n* = 6) shows an increased IL-6 mRNA relative expression in Aβ rats sacrificed at 18 h, 72 h, 7 days and 30 days. Cytokine levels (pg/mL) are presented as mean with SEM bars. IL-6 mRNA expression was normalized with β-actin. Data are expressed in average fold change with SEM bars. Significance level * *p* < 0.05, ** *p* < 0.01, and *** *p* < 0.001 are marked.

Glial cell activation by AβO leads to the release of several inflammatory mediators. For this reason, we quantified the proinflammatory cytokines IL-1β, IL-6, and the anti-inflammatory IL-10 in rat hippocampus homogenates using ELISA. No significant differences of IL-1β and IL-10 between the rats microinjected with AβO nor saline solution at 18 h, 72 h, and 7 and 15 days (Figure 8A) were observed. An increase of release of the three cytokines (*p* < 0.01 for IL-1β and IL-10, *p* < 0.05 for IL-6) was noticed in AβO rats sacrificed at day 30. 

IL-6 levels in AβO-injected rats decreased in 18 h, 72 h, and 7 d. Next, we evaluated IL-6 transcripts by qPCR (Figure 8B). In contrast to the protein expression, IL-6 mRNA levels were higher in oligomer-injected rats after 18 h (*p* < 0.001), 72 h (*p* < 0.001), 7 days (*p* < 0.001), and 30 days (*p* < 0.01).

### 2.5. Aβ Oligomers Promotion of STAT3 Expression in Glial Cells

**Figure 9:**
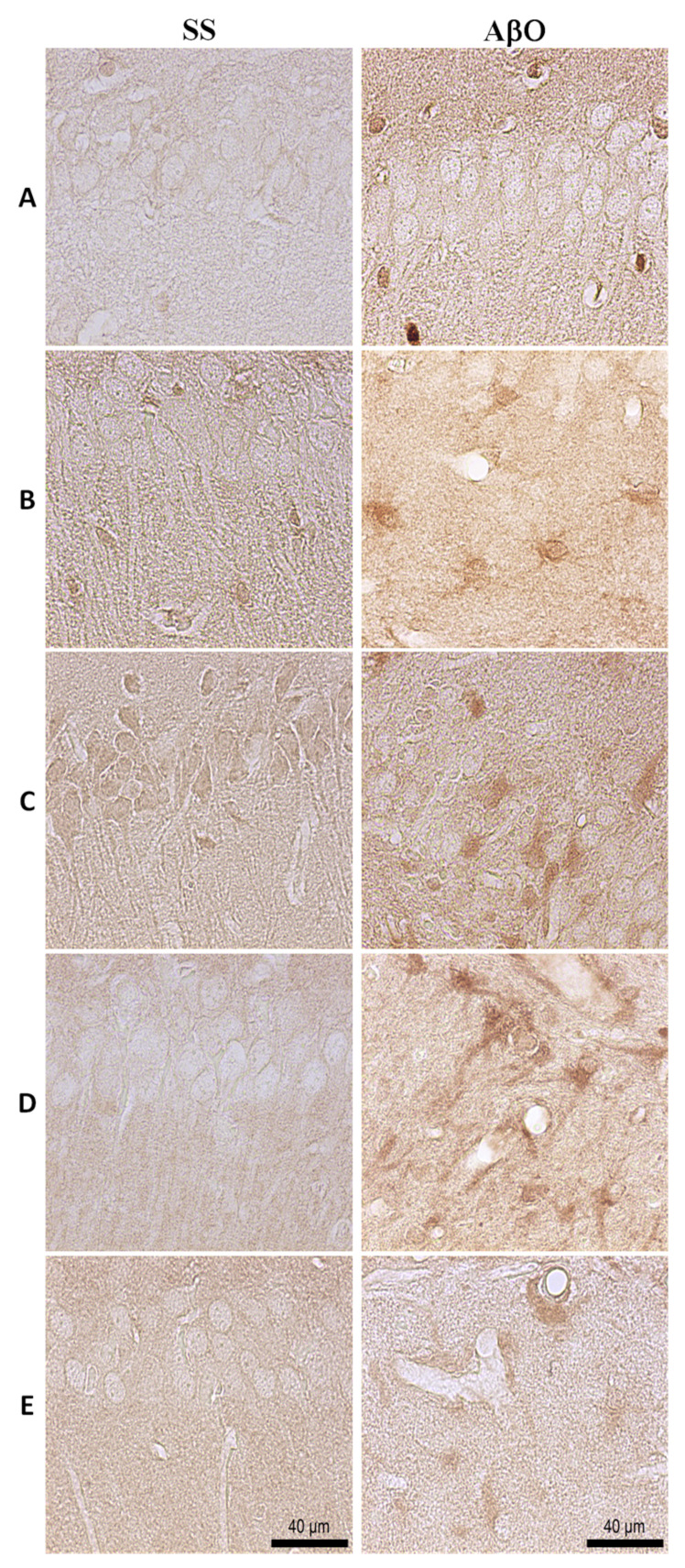
STAT3 expression in the hippocampus. Representative IHC (40×) images (scale bar = 40 µm), show a higher expression of STAT3 mediated by AβO at (**A**) 18 h, (**B**) 72 h, (**C**) 7 d, (**D**) 15 d, and (**E**) 30 d, after microinjection. At 72 h, STAT3 positive cells with glial morphology were identified, with a hypertrophic morphology demarked at day 7 and 15. Some of these cells showed STAT3 nuclear expression.

We next aimed to identify if the IL-6 signaling downstream transcription factor STAT3 was activated. Previously has been reported that STAT3 participate inducing neuronal death [28], for this we evaluated total STAT3 expression by IHC. After 18 h from sacrifice small STAT3 positive cells were distributed in hippocampus and cortex parenchyma, in rats microinjected with saline solution or AβO (Figure 9A). Most of these cells display an infiltrate-like morphology with nuclear immunoreactivity, being slightly higher in AβO rats.

At 72 h after microinjection (Figure 9B), an evident STAT3 expression in cells with glial morphology was observed in the hippocampus of AβO group; some of these cells presented swollen cytoplasm and immunoreactivity in the nuclei. In the respective control group, only a few positive infiltrate-like cells were observed.

The 7-day control group rats (Figure 9C) exhibited neuronal STAT3 expression with cytoplasm distribution. By contrast, STAT3 in AβO rats was restricted to glial cells, which displayed clearer activated morphology and nuclear immunostaining than the previous time.

High IL-6 mRNA levels (Figure 8B) correlate with an increased glial STAT3 expression in AβO rats sacrificed at 18 h, 72 h and 7 days (Figure 9); these results suggest JAK-STAT3 pathway activation, although cytokine levels were low at the same times (Figure 8A).

Furthermore, results from AβO rats at day 15 suggest a high glial STAT3 expression in CA1 and DG, similarly with day 7. These cells display a hypertrophic morphology, not presented by saline solution rats (Figure 9D).

Few STAT3 glial cells with resting morphology were detected in the cortex of control rats at day 30 (Figure 9E). AβO-injected rats exhibited more STAT3 glial cells in the hippocampus, although some of them had an activated morphology and nuclear staining, the latter was decreased in comparison to AβO rats sacrificed at previous times. 

**Figure 10:**
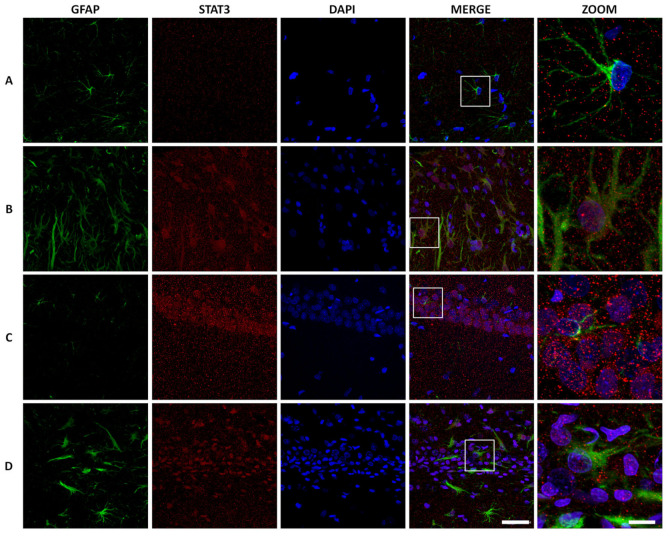
STAT3 expression in astrocytes. Immunofluorescence representative 63× images with digital zoom (scale bar 63× images = 40 µm, zoom = 10 µm) show an increased nuclear STAT3 expression (Alexa 555) in astrocytes mediated by AβO (Alexa 488), 72 h and 7 days after microinjection. The 7 day-saline solution control showed a cytoplasm STAT3 expression as previously observed in IHC.

To identify STAT3 localization, we performed immunofluorescence at 72 h and 7 days, where we detected consistent neuronal death. The vast majority of STAT3 positive cells were astrocytes and presented nuclear stain (Figure 10). Interestingly, on day 7, we detected STAT3 nuclear staining in some neurons of the CA1 region, which was not observed before with IHC. Certainly, STAT3 cytoplasmic expression in the control group rats was confirmed.

### 2.6. STAT3 Inhibition Assay in Hippocampal Primary Culture

An inhibition assay was conducted to evaluate the STAT3 expression in neurons and astrocytes exposed to AβO. Considering our previous results, we decided to expose a primary mixed hippocampal culture for 72 h to a 20 µM AβO solution from the human peptide. 

**Figure A1:**
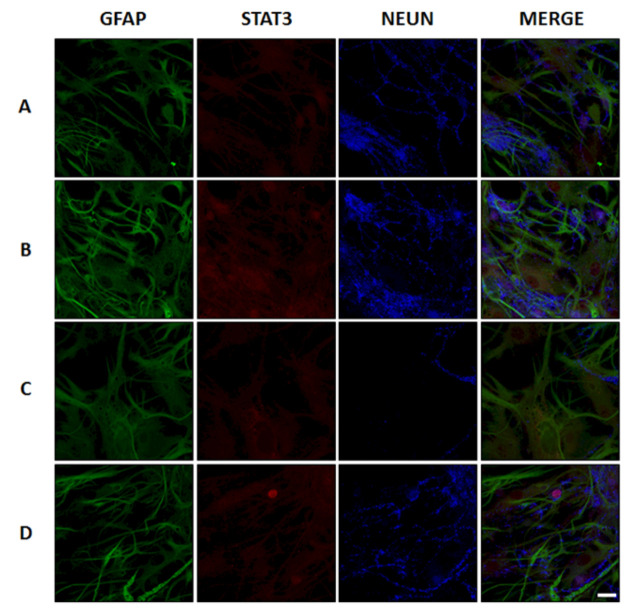
AG490 (25 µM) inhibition assay. Immunofluorescence representative 40× images (scale bar 40× images = 40 µm) of inhibition assay in primary hippocampal cultures, after 72 h of exposition to (**A**) Vehicle, (**B**) AG490 25 µM, (**C**) AβO 20 µM, and (**D**) AG490+AβO. Total STAT3 expression (Alexa 555), astrocytes with GFAP (Alexa 488), and neurons with NeuN (Cy5) were monitored. Although AG490 did not promote evident changes in STAT3 expression, it seems to prevent neuron loss in cultures exposed to AβO.

**Figure A2:**
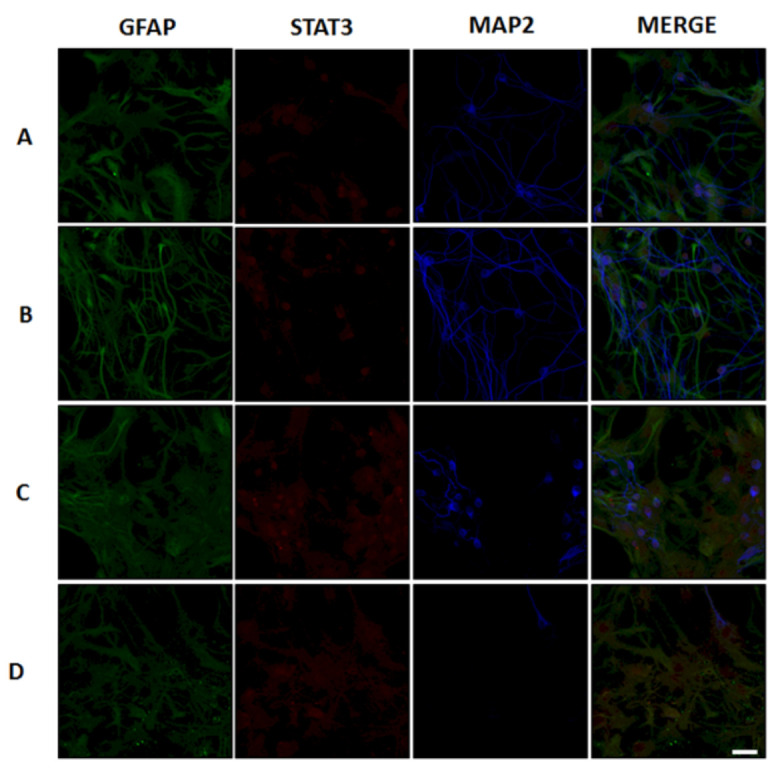
AG490 inhibition assay (50 µM). Immunofluorescence representative 40× images (scale bar 40× images = 40 µm) of inhibition assay in primary hippocampal cultures, after 72 h of exposition to (**A**) Vehicle, (**B**) AG490 50 µM, (**C**) AβO 20 µM, and (**D**) AG490+AβO. Total STAT3 expression (Alexa 555), astrocytes with GFAP (Alexa 488), and neurons with MAP2 (Cy5) were identified. The use of AG490 at 50 µM decreased the number of astrocytes and the neuron viability.

We initially used AG490 (25 and 50 µM), a JAK2 inhibitor, without detecting significant changes in total STAT3 expression in cultures both exposed to AβO and to AG490 25 µM+AβO (Figure A1). However, with the AG490 25 µM+AβO treatment, we observed a decrease in activated astrocyte morphology and the presence of more mature neurons compared to AβO. Although the inhibitor at 50 µM (Figure A2) promoted an evident reduction of the expression of total STAT3 in the cells exposed to AβO and the change in astrocyte’s morphology was also detected, the number of neurons was lesser than in the culture only exposed to AβO.

**Figure 11:**
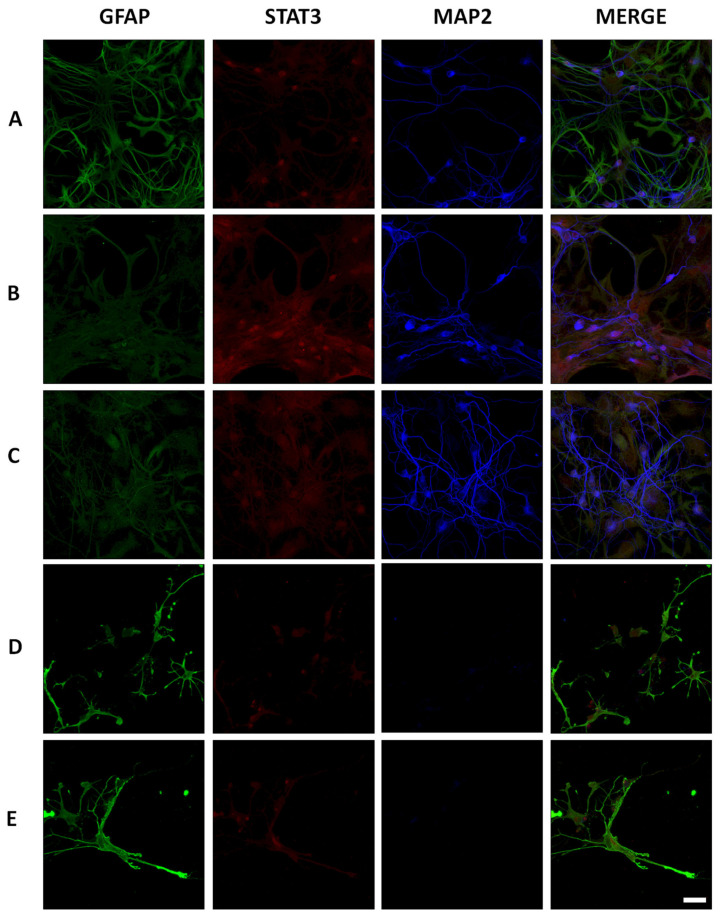
Stattic effect on primary hippocampal culture. Immunofluorescence representative 40× images (Scale bar 40× images = 40 µm) of Stattic inhibition probe in the concentrations 5 (**C**), 10 (**D**), and 20 µM (**E**) were contrasted to vehicle (**A**) and AβO (**B**) treatments. At the low-dose Stattic concentration, total STAT3 (Alexa 555) expression decreased in astrocytes (Alexa 488) and neurons (Cy5) compared with culture exposed to AβO. High doses of Stattic (D, E) promoted a decrease in astrocytes and neuron survival.

Next, we decided to probe Stattic, a small-molecule inhibitor of STAT3 expression [33] at different dilutions (5, 10, and 20 µM) to determine maximal inhibition concentration (Figure 11). Stattic inhibited STAT3 expression in a dose-dependent manner (Figure 11C–E), at 10 and 20 µM, expression of STAT3 increased in neurons and astrocytes of AβO-injected rats (Figure 11B). Noteworthy, at 10 and 20 µM we observed a deleterious effect in neuronal (MAP2-positive cells) and astrocytes (GFAP-positive cells) survival. For this reason, we used Stattic at 5 µM to probe the effect under AβO exposition.

**Figure 12:**
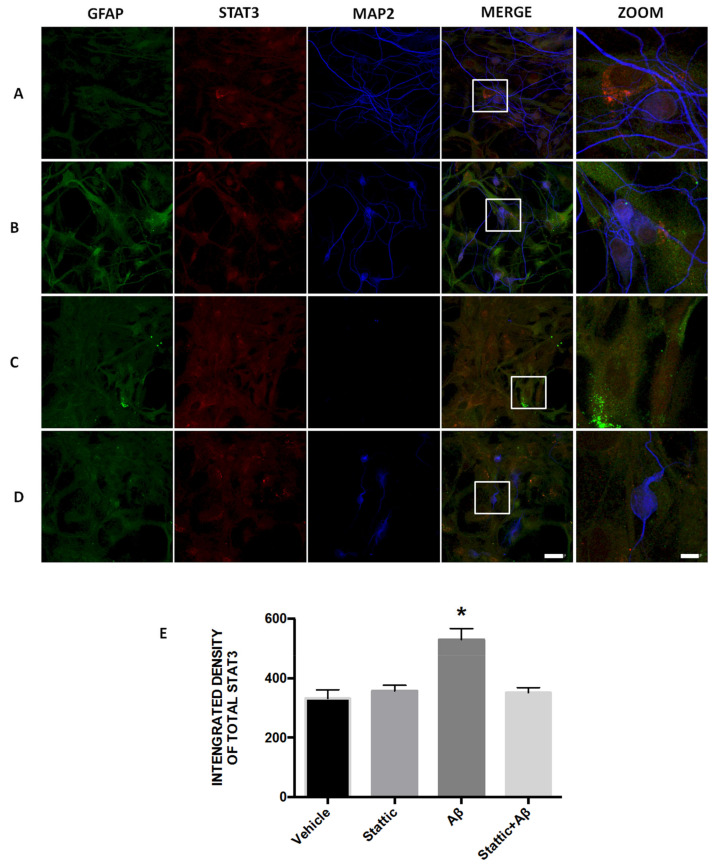
STAT3 inhibition assay. Immunofluorescence representative 40x images with digital zoom (scale bar 40× images = 40 µm, zoom = 10 µm) of inhibition assay in primary hippocampal cultures, before 72 h of exposition to (**A**) vehicle, (**B**) Stattic 5 µM, (**C**) AβO 20 µM, and (**D**) Stattic+AβO. Total STAT3 expression (Alexa 555), astrocytes with GFAP (Alexa 488), and neurons with MAP2 (cy5) were monitored. (**E**) Integrated density of Total STAT3 immunoreactivity. Data are means ± SEM (*n* = 4). One-way ANOVA followed by Tukey’s post hoc test (* *p* < 0.05).

**Figure A3:**
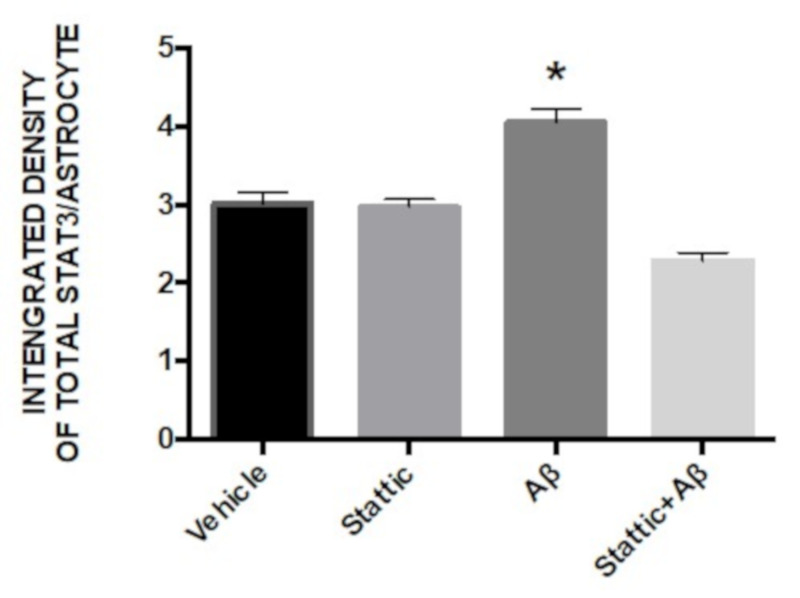
Integrated density of Total STAT3 immunoreactivity/astrocytes. Data are means ± SEM (*n* = 4, 4 astrocytes). One-way ANOVA followed by Tukey’s post hoc test (* *p* < 0.05).

On 5 µM Stattic incubation, STAT3 (Figure 12B) was expressed in all cells, which preserved the morphology and the number of astrocytes (GFAP) and neurons (MAP2), similar to the vehicle (Figure 12A). In contrast, AβO exposure favored neuronal death and an increase in STAT3 expression in GFAP positive cells (Figure 12C). When the inhibitor was added before AβO (Figure 12D), it promoted a decrease of STAT3 expression in astrocytes. In addition to these changes, the inhibitor prevented the death of neurons (Figure A3). A higher integrated density was detected in cell cultures treated with AβO (*p* < 0.05) in comparison to vehicle, Stattic, and Stattic+AβO (Figure 12E). The Astrocytes of AβO exposed cultures (Figure A3) presented a higher integrated density of total STAT3 immunoreactivity than the other treatments (*p* < 0.05). In contrast, in neurons, we identified a significant reduction in STAT3 integrated density in AβO treated cultures (*p* < 0.05).

## 3. Discussion 

Several AD genetic models have been developed to investigate Aβ toxicity. It has been thought that only Aβ fibrils or plaques could induce neuronal death in transgenic mice [34]. However, some authors have demonstrated the relevance of non-transgenic models based in an intracerebroventricular (ICV) or intrahippocampal AβO microinjection using non-human primates and rodents [35,36,37,38,39,40,41,42,43]. The main advantage of these models is the time it takes for damage to be apparent. We chose this approach to study the molecular mechanisms involved AβO toxicity, using a rat intrahippocampal microinjection model. Detrimental effects were observed at 24 h of a single microinjection of the Aβ 1-42 oligomers in the rat retrosplenial cortex compared with controls rats, albeit the effect was restricted to the injured region [41]. In this model, a single injection of AβO in CA1 activated the degeneration process, which continues until 30 days after surgery, which allowed the evaluation of the chronic damage generated by AβO. Neuronal degeneration was lower in the control group despite receiving the same mechanic damage, demonstrating the potential of AβO to induce neuronal death through several extracellular and intracellular mechanisms [15,16,44,45]. 

Another way of AβO-induced neuronal death comes from astrocytes and microglia proliferation and activation. It has been proposed that both cells could mediate the Aβ aggregates phagocytosis, particularly fibrils, displaying a protective role. In contrast, AβO could induce a deleterious inflammatory microenvironment that exacerbates neurodegeneration, with impaired phagocytic activity [18,19,25,46,47]. The method of Aβ oligomerization starting from the synthetic Aβ 1-42 human peptide used in the present project was extensively documented for the obtention of soluble globular oligomers [48], which were corroborated by electron microscopy (data not shown). Conducting the oligomer size analysis in a gradient 10–20% SDS-PAGE allowed us to identify low molecular weight oligomers (<17 KDa) and monomers (data not shown). Nonetheless, we cannot discard the presence of high molecular weight oligomers or protofibrils. In this sense, it has been controversially discussed that SDS affects the oligomerization state of Aβ. For this reason, in future works, it will be necessary to conduct a photo-induced cross-linking of unmodified proteins previous to SDS-PAGE or realize an oligomer characterization by ion mobility coupled to electrospray ionization mass spectrometry [49,50].

Results show that our AβO preparation promoted astrocyte and microglia proliferation. The relation between glial activation and neurotoxicity was the presence of hypertrophic astrocytes and microglial cells with swollen or amoeboid morphology, and neuronal loss. The highest number of activated glial cells was observed at 72 h, correlating with extensive degeneration visualized in DG with the FJB staining. Interestingly, the proportion of activated astrocytes was higher than microglia, though this myeloid cell was located near to neuron loss spaces. Several reports have suggested that activated astrocytes could contribute to Aβ production and aggregation [51,52]. A recent work elucidated that astrocytes stimulated by AβO undergo an increase of BACE1 and ApoE expression [46]. 

The ultrastructural analysis allowed us to validate the close relation between neuronal alterations and glial activation. At 18 h, neuropil alterations and the low number of neurons correlated with the increased neurodegeneration observed by FJB staining. Also, significant changes in neuron morphology are reflected by cytoplasm disruption and loss of organelles, accompanied by surrounding hypertrophic astrocytes. Moreover, a high degree of disruption of myelin sheaths also suggests axon degeneration. Changes of the inner membrane and cristae of mitochondria are synonymous of mitochondrial dysfunction. Both abnormalities induced by Aβ aggregates have been previously described [53,54]. Spaces of neuronal loss were associated with activated microglial cells containing organelle debris inside vacuoles, remarking the role of microglial cells in AβO-mediated neurotoxicity. 

The presence of amyloid-like fibrils, as well as of intracellular AβO, should be confirmed in future experiments of immunoelectron microscopy in the AβO rats. There is a high probability that a part of the AβO preparation could form fibrils due to the physiological temperature and pH conditions of rat brain tissue. This is supported by the fact that we were able to identify activated microglia near those fibrils and that they were not observed at later times, probably due to phagocytosis.

Although at day 30 in AβO rats, a reduction in cytoplasmic alteration was observed, Aβ continued exerting neurotoxicity effects and promoting inflammation, due to the presence of axonal degeneration, as well as microglia with amoeboid morphology and organelle phagocytosis, suggesting that Aβ is capable of inducing chronic neuronal death, altering DG neurogenesis, and decreasing cell proliferation [55].

Considering the presence of glial activation in all the evaluated times, we quantified IL-1β, IL-6, and IL-10, which have been associated with beneficial or detrimental activity related to Aβ aggregates neurotoxicity [56,57,58]. In our model, the AβO or saline solution groups brought under a mechanical injury that promoted glial activation. However, this damage was lesser in the control group, which could explain why we did not observe significant differences in IL-10 and IL-1β levels in both groups at acute times.

About the IL-1β and IL-6 significant increase in AβO rats at day 30, we suggest these proinflammatory cytokines mostly derive from the high number of activated microglial cells. Previous reports have indicated the IL-1β role in the induction of glial activation and neuron damage [59]. Despite IL-1β overexpression may favor Aβ clearance [57], it may also contribute to tau phosphorylation [60]. A similar effect of AβO clearance was described for IL-6 by mediating gliosis [51], extending the neuron loss induced by the presence of Aβ and the overstimulation of NMDAR [61]. It is possible that the release of these cytokines would be related with the absence extracellular amyloid-like fibrils as observed at 72 h and 7d. Even though, this is not suggestive of Aβ clearance from the hippocampus and we consider this to be an important issue to address in the near future. 

IL-10 is a cytokine, which reduces inflammation and apoptosis. For this reason, it has been proposed as a possible therapeutic target in several CNS disorders, such as AD [62]. However, IL-10 overexpression in APP transgenic mice or its deletion in APP/PS1 mice revealed that the anti-inflammatory effects would detrimental by reducing AβO clearance by microglial cells [56,63] because the effectiveness of this process is promoted by an inflammatory microenvironment. 

In the present study, IL-10 expression was observed in AβO rats after 30 days and could be favoring neuron density as well as decreased neuron alterations aforementioned. Despite IL-10 expression, confocal an electron microscopy showed that phagocytosis was not diminished in microglia, which may be due to high levels of IL-1β and IL-6. This data suggests the need for a balance in the cytokine microenvironment, rather than a predominant inflammatory profile. 

IL-1 and IL-6 are proinflammatory cytokines; however, the role of both plays in the inflammatory process may be different. It is known that IL-6 is a pleiotropic cytokine. Reported studies indicate that the overexpression of IL-6 in CNS generates the presence of reactive gliosis that can lead to neurodegeneration. On the other hand, it is known that IL-10 has a fundamental role in limiting chronic and acute inflammation. However, under pathological conditions, it is known that the levels of pro- and anti-inflammatory cytokine may be elevated. This has been demonstrated in the CSF of patients with AD [64]. However, these findings have failed to elucidate whether the presence of Aβ is associated with a poor response of innate immunity, which could accelerate the cascade of pathological events, or if neuroinflammation is due to a mechanism of rescue that attempts to eliminate the accumulation of toxic amyloid species, through the activation of glial cells. This alteration of the immune response would prevent the elimination of accumulated peptides and lead to altered production of cytokines, which favor the neurodegenerative process. Moreover, the presence of pro- and anti-inflammatory molecules would be the result of a change in the cytokine profile, closely related to the pathophysiology of the disease, in the particular case of this model, with the presence of AβO aggregates.

An interesting finding of our experiments was a reduction of IL-6 in AβO rats from 18 h to 7 days. For this reason, we contrasted these results with relative IL-6 mRNA expression, detecting high mRNA levels in AβO administrated rats compared with the control group. 

It has been documented that the underestimation of cytokine production is possibly due to cellular consumption in cytokine quantification by ELISA [65], which could involve processes of cytokine binding to its receptor or protein degradation. In this sense, some molecular mediators (IL-6, IL-1β, TNFα, among others) display two receptor types: a transmembranal receptor, which activates the classical signaling pathway; and a soluble receptor, which induces the trans-signaling. If the cytokine is bound to its soluble receptor in a biological sample, it typically cannot be quantified with an immunoassay-based measurement [66]. 

We think that 18 h to 7 days, exists the possibility that IL-6 could be bound to its soluble receptor, making difficult the protein determination by ELISA multiplex assay. Considering that IL-6 was activating the trans-signaling pathway we identified STAT3 expression, a key transcription factor of the IL-6 signaling. A previous report of Aβ aggregated microinjection in mice and cell culture exposition showed that Aβ induces STAT3 activation through tyrosine phosphorylation (p-Tyr STAT3), leading to apoptosis only in neurons [28]. Some oligodendrocytes and infiltrates were the first STAT3 positive cells detected after microinjection, probably as part of the inflammatory response initiated after the needle injury. At the same time, AβO promoted a slight increase in STAT3 nuclear expression. Although only total STAT3 expression was identified, nuclear accumulation of this factor is a signal of activation [30,67]. 

After 72 h, when AβO mediated neurodegeneration and glial activation is observed, a moderate STAT3 glial increased expression was evident. The predominant cell population consisted of astrocytes exhibiting nuclear activation. 

Neurodegeneration persisted closely to glial activation in AβO rats on day 7, where STAT3 expression increased in glial cells with activated morphology; some of them displayed nuclear expression. Moreover, complementary immunofluorescence exposed nuclear STAT3 expression in neurons of the CA1 region. In contrast, cytoplasmic STAT3 neuronal expression was detected in control rats. This should be studied in the future due to its possible relevance in neuronal recovery after traumatic injury. Supposing, IL-6 trans-signaling in previous acute times, it would justify the high STAT3 astrocyte expression, mainly at day 7 after AβO administration. STAT3 expression remained 15 days after surgery correlating with high glial activation and neuronal death. Besides, the classical IL-6 signaling pathway could induce anti-inflammation and a proinflammatory response by trans-signaling; it has been recently studied because of its implication in neurodegenerative disorders, such as amyloid pathology in AD [68,69]. Cytoplasmic STAT3 expression disappeared on day 15 in controls, but AβO rats exhibited nuclear STAT3 expression in hypertrophic glial cells, correlating with persistent neuronal density reduction.

STAT3 is an important transcription factor activated by stimulation of several proinflammatory (IL-1, TNFα, IL-6) and anti-inflammatory (IL-10) molecular mediators. In the CNS, controversial functions of STAT3 has been reported, which could be due to a cell-specific response or the inflammatory microenvironment. For example, STAT3 has been involved in axon regeneration [70]. Besides, STAT3 activation could modulate synaptic plasticity trough induction of NMDAR-long term depression [71]. However, inflammation it has been recognized as an inductor of STAT3 derived from glial activation, which could promote different neuropathological processes, such as neurogenesis impairment and apoptosis [72]. 

Regarding apoptosis, STAT3 activation by proinflammatory cytokines enhances cytochrome C release and caspase activation [73]. In an epilepsy rat model, it is known that STAT3 is activated after status epilepticus. The administration of kainic acid significantly increases STAT3 levels linked to increased IL-1 production. The inhibition of STAT3 by WP1066 reduces IL-1 levels, Iba1 expression, and neuronal death that leads to the neurodegeneration observed [74]. With respect to microinjection of Aβ in animal models, the potential as STAT3 activator in neurons has been explored, favoring the transcription of genes linked to oxidative damage (as iNOS) that could be responsible for inducing apoptosis [28]. In our animal model, the expression of STAT3 is observed in acute times and correlated with the increase in neurodegeneration levels evidenced by FJB staining. We have considered that STAT3 increasing expression in AβO rats could mediate astrocyte [75] and microglial activation, as proposed previously [31]. Nuclear STAT3 localization in astrocytes and neurons could stimulate different target genes related to inflammation and neurotoxicity. STAT3 activation was previously detected only in neurons and related to apoptosis [28].

To clarify the JAK2/STAT3 role, we designed an inhibition assay in rat primary hippocampal mixed culture, using a JAK2 (AG490) and a STAT3 specific inhibitor (Stattic). Cultures were exposed to a cytotoxic concentration of AβO for 72 h, a time that course with high neurodegeneration in the animal model. Both assays obtained results suggesting that low-moderate inhibition of the JAK2/STAT3 pathway decreased glial activation, which helped to prevent a neuron death promoted by AβO. However, when we tried to increase the inhibition state of STAT3, the results showed a reduced survival of neurons and astrocytes. The above pointed out that the JAK2/STAT3 pathway plays an essential role in AβO toxicity since it promotes the reactivity of astrocytes, which could secrete different inflammatory mediators that lead neurons to the neurodegeneration process. 

In this sense, studies have consistently demonstrated the pathogenic JAK2/STAT3 pathway activation in glial cells is primed by the presence of aggregated Aβ. The JAK2 stimulation in AD transgenic mice (APP/PS1 or 3xtgAD) indicated that STAT3 activation promotes astrogliosis and synaptic dysfunction. Conversely, astrocyte-specific inhibition of the JAK2/STAT3 pathway in APP/PS1 mice control astrocyte reactivity restores synaptic deficits, and produces a Aβ deposition reduction, not linked to a microglial phagocytosis increase [30]. Moreover, a developing of an astrocyte conditional STAT3 knockout in APP/PS1 transgenic mice leads to an anti-inflammatory profile, which could mediate Aβ clearance by microglial cells and reduce cognitive decline [29]. Finally, another recent study in astrocyte primary culture exposed with AβO and treated with Stattic (1 µM) showed that STAT3 is responsible for maintained reactive state of astrocytes, and dismisses changes in ERK and NF-kB. Also, the oral administration of this compound in the 5xFAD mice reduced the STAT3 activation in astrocytes, correlating with an improvement in cognitive function [76]. 

The JAK/STAT3 inhibition is a promising strategy to modulate glial reactivity to favor neuron survival under AβO insults. Nonetheless, as observed in our assays, high or total inhibition of the JAK2/STAT3 pathway could induce neurodegeneration due to its crucial role in cell proliferation, axon regeneration, and synaptic plasticity. Thus, the ideal strategy would be to modulate the pathway only in astrocytes, as stated by the previously mentioned experiments [29,30]. However, we know that one difference between our microinjection model and pathology, as AD is the constant Aβ aggregation, which induces a persistent inflammatory response and progressive neurodegeneration. Also, the recovery of AβO 30-day rats was partial compared to their respective control group, possibly related to the difference between STAT3 expression in both groups. The increased transcription factor expression and activation in AβO rats at day 30 could be favored by IL-6 and IL-10, two JAK/STAT3 inducers [77]. 

Future studies should clarify both cytokines’ role in the dynamic of STAT3 activation, considering that both resulted increase on day 30. Another issue that should be explained is the possibility of density recovery due to the reduction of Aβaggregates. If so, it would be interesting to evaluate the administration of a second AβO dose.

## 4. Materials and Methods 

### 4.1. Aβ Oligomers

*A*βO were obtained from synthetic Aβ 1-42 peptide (AnaSpec San Jose, CA, USA AS-20276) using a method previously described [48]. The peptide was diluted with 1,1,1,3,3,3-hexafluoro-2-propanol (Sigma-Aldrich Merck KGaA, Darmstadt, Germany H-8508) and incubated 1 h at room temperature followed by 15 min to 4 °C. The solvent was evaporated overnight, and the pellet was suspended in Dimethyl sulfoxide (Sigma-Aldrich Merck KGaA, Darmstadt, Germany) to get a 5 mM solution. Finally, to obtain the oligomer forms aliquots of 100 µM of the peptide were prepared with sterile phosphate-buffered saline 1× and incubated to 4 °C for 24 h. A simple transmission electron microscopy (TEM; FEI-TecnaiBioTWIN, Hillsboro, OR, USA) analysis of 5 µL of this solution was conducted to corroborate the presence of globular oligomers and the absence of fibrils. Also, low-molecular weight oligomers (<17 KDa) were corroborated with western blot, using the Aβ antibody 6E10 (MAB1560, Chemicon, Merck Millipore, Massachusetts, USA) (data not shown).

### 4.2. Animal Model

Male Wistar rats (Rattus novergicus) aged 12–14 weeks and 230–250 *g* of body weight were purchased from Bioinvert^®^ bioterium, Estado de México, México (SENASICA AUT-B-A-1016-028). Animal experiments were conducted according the Official Mexican Norms (NOM-062-ZOO-1999 and NOM-033-SAG/ZOO-2014) and the Code of Practice for the Housing and Care of Animals Used in Scientific Procedures. Also, the experimental protocol (no. 113/09) was approved by the Ethics Committee of National Neurology and Neurosurgery Institute “Manuel Velasco Suárez” (April 2009).

Housing conditions were 22–24 °C of temperature, 50–60% humidity, 12/12 h light/dark cycle, water and food ad libitum. Rats were anesthetized with an intramuscular injection of xylazine (12 mg/kg) and ketamine (85 mg/kg), pain were managed with tramadol. A bilateral microinjection was performed in a stereotaxic surgery apparatus (Stoelting, Sheboygan, USA) in the coordinates corresponding to CA1 hippocampus region (*V*:-0.30 mm, *L*: ± 0.25 mm y *AP*: −0.42 mm from bregma) [78]. The rate of infusion pump was 12 µL/min and we used a 30 G dental needle, which was removed 5 min after the injection. In general, we create two study groups, the control group was injected with vehicle and the other group, conformed by rats microinjected with AβO. In each treatment we design a temporal study, considering subgroups of rats that was sacrificed in acute (18 and 72 h) and chronic (7, 15, and 30 days) times after surgery.

### 4.3. Immunohistochemistry and Immunofluorescence

Rats were anesthetized as previously mentioned and perfused with cold and sterile saline solution 0.9%, followed by 4% Phosphate-Buffered Paraformaldehyde. Brains were removed and stored for 1 week in a solution with 4% Phosphate-Buffered Paraformaldehyde and 4% of sucrose. Brain hemispheres were divided and dehydrated in gradual alcohols (70%, 96%, and 100%), followed by xylene treatment and paraffin embedded. 8 µm-thick sagittal serial slices were obtained, and the slides were deparaffinized and hydrated. Antigen recovery was performed with 0.1 M citrate buffer, pH 6 and 0.2% Triton at 60 °C for 12 min. For IHC, endogenous peroxidase was inactivated with blocking reagent (Dako- Agilent Santa Clara, CA 95051 USA, S2003) for 10 min. Additionally, the tissue was incubated with bovine serum albumin (BSA) 2%-Triton 0.2% for 15 min. Slides were blocked with Background Sniper (Biocare Medical, California USA, BS966) for 15 min. Primary antibodies were diluted in BSA 1% and incubated at 4 °C overnight, followed by incubation Mach2 Universal HRP-Polymer Detection (Biocare Medical, California USA, M2U522) for 30 min. 3, 3′-diaminobenzidine (Dako K3468) was used for color developing. Slides were mounted and analyzed on a Leica DM LS microscope (Leica Microsystems, Wetzlar, Germany). STAT-3 images were captured with the ScanScope CS digital processor (Aperio, San Diego, CA, USA).

Primary antibodies used for IHC were mouse monoclonal anti-NeuN (1:250, Millipore, MAB377), rabbit polyclonal anti-STAT3 (1:1000, Santa Cruz Biotechnology, Santa Cruz, California. USA, Sc-482).

After antigen retrieval in immunofluorescence, sections were permeabilized as we previously mentioned and blocked in 2% BSA for 30 min. Primary antibodies diluted in BSA 1% were incubated at 4 °C overnight, followed by secondary antibody incubation for 1 h at room temperature. In some cases, nuclei were stained with 4′, 6-diamidino-2-phenylindole (DAPI, Invitrogen, D1306) for 5 min. Slides were mounted with Vectashield antifade mounting medium (Vector Laboratories, California, USA, H-1000). 

Images were captured on a Leica TCS SP8 confocal microscope and processed with LAS AF software (Leica Microsystems). Primary antibodies used in IF were mouse monoclonal anti-NeuN (1:200, Millipore, Merck KGaA, Darmstadt, Germany MAB377), chicken polyclonal anti-GFAP (1:1000, Abcam, Cambridge UK, ab4674), rabbit polyclonal anti-GFAP (1:150, Genetex, California USA, GTX16997), rabbit polyclonal anti-Iba1 (1:300, Genetex, California USA, GTX100042), and anti-STAT3. The secondary antibodies used were goat anti-chicken IgY Alexa Fluor 488 (1:300, Abcam, ab150173), goat anti-mouse IgG Cy5 (1:150, Zymed, Thermo Fisher Scientific, Massachusetts, USA, 816516), anti-rabbit IgG Alexa 555 (1:300, Cell Signaling, Massachusetts, USA, 4413S). 

### 4.4. FluoroJade B Staining

FJB was used to stain brain cells under degeneration [79]. Tissue 8 µm-thick sections were deparaffinized and hydrated. Slides were incubated in 80% EtOH-1% NaOH for 5 min, followed by 2 min in 70% ethanol and 2 min in distilled water. Sections were immersed in 0.06% K_2_MnO_4_ for 20 min, after distilled water washes, slides were incubated at 4 °C overnight in 0.001% Fluoro-Jade B (Millipore, AG310) previously diluted in 0.1% acetic acid [80]. Slides were dried, immersed in xylene and mounted with Eco-Mount (Biocare Medical, EM897L). Images were captured at 40× magnification on a Leica DM LS microscope with epifluorescence (Leica Microsystems, Wetzlar, Germany).

### 4.5. Quantification of NeuN and FluoroJade B

Mature neurons were identified with Anti-NeuN antibody in control and AβO microinjected rats sacrificed on days 15 and 30 after stereotaxic surgery (*n* = 4, per group and time). For degenerated neurons, the FJB staining was evaluated in control and AβO groups at 18 h, 72 h, and 7 days post-surgery (*n* = 4, per group and time). 

Quantification of NeuN and FJB positive cells was realized with 8 µm-thick sagittal sections obtained from injury (2.5–2.7 mm lateral from bregma), one out every 10 sections was evaluated. Cell count was performed on the Leica Application Suite software version 4.0 (Leica Microsystems, Wetzlar, Germany) on a 265 × 365 µm frame at 40× magnification, analyzing 3 adjacent fields on CA1 region and the same for dentate gyrus. The experimental groups were blinded in during the count and we considered positive cells with whole cell bodies within the counting frame. Average of positive cells per mm^2^ were reported [80,81]. 

### 4.6. Electron Microscopy

Rats were perfused with 4% Paraformaldehyde-2.5% Glutaraldehyde in 0.1 M phosphate-buffered saline (pH 7.4). Brains were removed and post-fixed in the same solution for 24 h. Tissues were processed in a LEICA EM TP (Leica Microsystems, Vienna, Austria) and embedded in Epon resin (EMbed 812 Electron Microscopy Science, Kolkata, India, Cat. No.14120). A 90 nm slices were obtained, mounted in 200 mesh copper grids, and contrasted with 2% uranyl acetate and lead citrate. Images were captured on a TEM (FEI-TecnaiBioTWIN, Hillsboro, OR, USA) [82,83].

### 4.7. Cytokines Quantification

Rat hippocampus for each subgroup of study (*n* = 4, for each time and treatment evaluated) was dissected and homogenized in sterile phosphate-buffered saline containing 0.01% Triton X-100 and protease inhibitors (Sigma, P8340) [84], using the Sample Grinding Kit (Amersham Bioscience, Merck KGaA, Darmstadt, Germany cat. no. 80-6483-37) and Vibra-Cell^™^ Ultrasonic Liquid Processors (Sonics & Materials Inc., Connecticut, USA, Cat. No. VCX13). Samples were centrifuged at 1000 *g* and 4 °C for 10 min, to assure remaining debris remove, the resulted supernatant was centrifuged at 20,000 *g* for 40 min at 4 °C [85]. The cytokines IL-6, IL-10, and IL-1β were quantified by an Enzyme-Linked ImmunoSorbent Assay (ELISA) using the MILLIPLEX^®^ MAP Rat Cytokine/Chemokine magnetic bead panel kit (Millipore), according to the supplier’s protocol (RECYTMAG-65K-03; Millipore, Temecula, CA, USA). The panel kit was reading with the LUMINEX MAGPIX^®^ detection system with xPONET^®^ software and the results were analyzed in MILLIPLEX^®^ Analyst version 5.1 (Millipore, Billerica, MA, USA). The cytokines sensitivity ranges were 2.4–10,000 pg/mL for IL-1*β*, 73.2–300,000 pg/mL for IL-6, and 7.3–30,000 pg/mL for IL-10.

### 4.8. RT-PCR

Total RNA was isolated from rat hippocampus (*n* = 4, for each time and treatment evaluated) with TRIzol^®^ reagent, considering the supplier’s protocol (Invitrogen Corporation, Carlsbad, CA, USA, cat. no.15596026). cDNA was synthesized from 500 ng of total RNA using AMPIGENE^®^ cDNA Synthesis kit (Enzo Life Sciences, ENZ-KIT106). The reverse-transcribed product was diluted five times and 2.0 μL of cDNA was mixed with 2× TaqMan Master Mix and 0.5 µL of 20× Gene Expression Assay, the reaction was conducted in 7500 Fast Real-Time PCR system (Applied Biosystems, Foster City, CA, USA). The TaqMan^®^ gene-specific probes (Applied Biosystems, Foster City, CA, USA) selected in this work were IL-6 (cat. no. Rn01410330_m1) and β-actin (cat. no. Rn00667869_m1). The 2^−*ΔΔ*Ct^ method was used to analyze the relative target gene expression respect the β-actin gene expression [86].

### 4.9. STAT3 Inhibition Assay in Cell Model

Primary mixed hippocampal cultures were obtained from 2–4 day old Wistar rats, with slight modifications to the method described previously by Osorio et al [87]. After 15 days of culture, a STAT3 inhibition assay was conducted. We used the small molecule Stattic (Santa Cruz Biotechnology, California, USA, Sc-202818) at different dilutions (5, 10, and 20 µM). Cultures were exposed to different treatments vehicle (DMSO), Stattic (5 µM), AβO (20 µM) or Stattic+AβO. We also realize two independent experiments using AG490 (Calbiochem, San Diego, CA, USA), a JAK2 inhibitor (25 µM and 50 µM). The inhibitors were added one day before AβO. After 72 h, cells were fixed with 4% Phosphate-Buffered Paraformaldehyde and immunofluorescence was performed with the next primary antibodies: mouse monoclonal anti-MAP2 (1:300, Millipore, MAB3418), mouse monoclonal anti-NeuN, rabbit polyclonal anti-STAT3 (1:100 Novus, NBP2-67432), chicken polyclonal anti-GFAP. Secondary antibodies were goat anti-chicken IgY Alexa Fluor 488, Goat Anti-Mouse IgG Cy5, goat anti-rabbit IgG Alexa 555 (1:300, Abcam, ab150086).

### 4.10. Statistics

Data were analyzed with MiniTab^®^ v. 19 software (State College, PA, USA), using a significance level (α) of 0.05. Before any statistical analysis, equality of variances was assessed by Levene’s test. 

Differences between five evaluated times for each treatment (SS or Aβ) were identified with a one-way ANOVA test and by specific comparisons. Subsequently, to determine statistical significance between the different study groups (SS versus Aβ) at equivalent time intervals, a *t*-test was conducted. The integrated density of total STAT3 was calculated in Fiji software (Fiji, ImageJ, Wayne Rasband National Institutes of Health, Bethesda, MD, USA) [88].

## 5. Conclusions

The temporal study of AβO rat intrahippocampal microinjection suggested, oligomers mediate indirect neurotoxicity and neurodegeneration through glial activation and the consequent inflammatory response. Based on our present results, AβO induces the JAK/STAT3 pathway stimulation in astrocytes promoting it activation state and in neurons favor surveillance. However, the constant activation of the pathway by AβO, leads to hypertrophic astrocytes, which may promote an inflammatory microenvironment and increased neurodegeneration. A therapy of JAK/STAT3 pathway inhibition, mainly directed to regulate astrocytes reactivity, could be an excellent strategy to diminish neuron death and cognitive decline in AD. The use of JAK/STAT3 inhibitors should be considered with caution because a high dosage would favor the neuronal death in addition to the generated by Aβ aggregates. Considering the direct relationship between inflammation and neurodegeneration at short times, it would be useful to try different drugs that modulate astrocyte activation states. The challenge is to probe if the return to a quiescent state could improve other AβO toxic effects such as mitochondrial dysfunction, LTP impairment, oxidative damage, membrane pore formation, neurogenesis, and plasticity impairments.

## Figures and Tables

**Figure A4:**
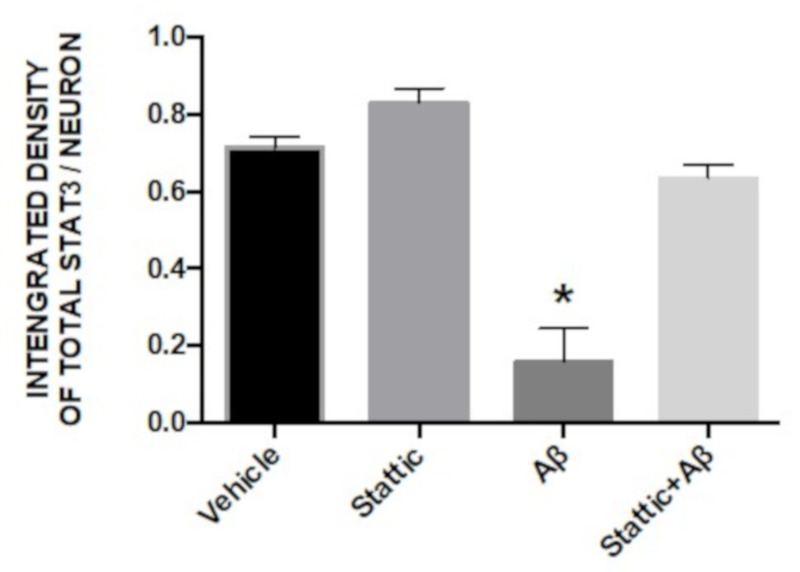
Integrated density of Total STAT3 immunoreactivity/neurons. Data are means ± SEM (*n* = 4, 3 neurons). One-way ANOVA followed by Tukey’s post hoc test (* *p* < 0.05).

## Abbreviations

AβO	Amyloid β oligomeric
JAK-STAT3	Janus kinase–signal transducer and activator of transcription 3
STAT3	Signal transducer and activator of transcription 3
IL-6	Interleukin 6
IL-10	Interleukin 10
AD	Alzheimer’s disease
NP	Neuritic Plaques
Aβ	Amyloid beta
Aβ40	Amyloid β 1-40
Aβ42	Amyloid β 1-42
AβPP	Amyloid β precursor protein
NMDAR	N-methyl-D-aspartic acid
IL-1β	Interleukin 1 beta
TNF-α	Tumor necrosis factor alpha
STAT	Signal transducer and activator of transcription
JAK	Janus kinase
JAK2	Janus kinase 2
FJB	Fluoro-Jade B
DG	Dentate gyrus
NeuN	Neuronal nuclei protein
GFAP	Glial fibrillary acidic protein
IBA1	Ionized calcium binding adaptor molecule 1
SS or S	Saline solution
Mt	Mitochondria
Ax	Axon
F	Fibril
M	Microglia
N	Neuron
A	Astrocyte
SEM	Standard error of the mean
Er	Endoplasmic reticulum
v	Vacuole
qPCR	Quantitative polymerase chain reaction
IHC	Immunohistochemistry
BACE1	β-secretase1
ApoE	Apolipoprotein E
JAK2-STAT3	Janus kinase 2 -Signal transducer and activator of transcription 3
3xtgAD	Triple transgenic Alzheimer disease mice
BSA	Bovine serum albumin
CNSICV	Central nervous systemIntracerebroventricular
